# An Open Standard for Camera Trap Data

**DOI:** 10.3897/BDJ.4.e10197

**Published:** 2016-12-06

**Authors:** Tavis Forrester, Tim O'Brien, Eric Fegraus, Patrick A Jansen, Jonathan Palmer, Roland Kays, Jorge Ahumada, Beth Stern, William McShea

**Affiliations:** ‡ Smithsonian Conservation Biology Institute, Front Royal, United States of America; § Oregon Dept. of Fish and Wildlife, La Grande, United States of America; | Wildlife Conservation Society, New York, United States of America; ¶ Conservation International, Arlington, United States of America; # Smithsonian Tropical Research Institute, Balboa, Panama; ¤ Wageningen University, Wageningen, Netherlands; « North Carolina State University, Raleigh, United States of America; » North Carolina Museum of Natural Sciences, Raleigh, United States of America; ˄ Smithsonian Data Center, Herndon, United States of America

**Keywords:** big data, biodiversity, camera trap, data repository, data schema

## Introduction

Accurately surveying and monitoring animal communities is an essential part of wildlife management and conservation (Nichols and Williams 2006). Monitoring mammals has been a continual challenge for wildlife researchers and managers because mammals are often nocturnal, occur at low densities, move over large areas, or actively avoid people (Long et al. 2008). Camera traps that record wildlife using heat or motion sensors provide a solution to this problem.

While camera traps have limits as a survey tool (Meek et al. 2015, Burton et al. 2015), the advantages as well as declining cost and increasing reliability have led to a rapid increase in the use of camera trapping as a survey method in the last decade (Rowcliffe and Carbone 2008, Burton et al. 2015). The scale of camera trapping research has also rapidly increased, with some researchers using hundreds of camera traps deployed over large geographic areas (e.g. 1.2 million images in Tanzania (Swanson et al. 2015), 2.6 million images in the eastern US (McShea et al. 2015), and 2.5 million images across tropical forests (http://www.teamnetwork.org/)). As no metadata standards for camera trapping have been adopted and researchers typically store their data on different platforms, sharing and aggregating camera trap data has been greatly impeded.

The difficulties of aggregating data among camera-trapping experts affiliated with a variety of organizations, including the Smithsonian Institution, the Wildlife Conservation Society, Conservation International, and the North Carolina Museum of Natural Sciences, directly led to the creation of this data standard. Researchers found that the use of different authorities for species names, inconsistent recording of habitat features, differing levels of recorded data regarding camera deployments, and most recently the tagging of single photos vs. photo bursts, all created problems when attempting to combine data. The lessons learned from large scale monitoring projects such as the Tropical Ecology Assessment and Monitoring network (TEAM) (Ahumada et al. 2013) and eMammal (McShea et al. 2015) were adapted to form the basis of a data standard that solves these problems and other issues of data reporting for camera trapping studies (Burton et al. 2015).

Here we present the Camera Trap Metadata Standard (CTMS). This data standard offers a common data format to facilitate data storage and sharing. The standard also provides a structure for researchers to manage their data. Finally the standard is an essential step to providing access to data through web services and other automated methods, an essential element of providing open access to research data (Hampton et al. 2012) and publishing data online. Most of the programs that have been developed to manage camera-trap photos and associated meta-data (Tobler 2007, Harris et al. 2010, Fegraus et al. 2011, Krishnappa and Turner 2014, Ivan and Newkirk 2016, Niedballa et al. 2016) organize data in a program specific way and store data locally, resulting in “dark data”, or data not available to other researchers or the public (Hampton et al. 2013). Dark data and incomplete data reporting have led to calls for open access to research data, especially research that is funded with government funds (Hampton et al. 2012, Hampton et al. 2013). The CTMS provides a framework for uploading camera trap data to data repositories and for creating a process for rapid data publication of camera trap data in the future.

## Description of the Data Standard

We categorize camera-trap data as hierarchical and in four levels (Project, Deployment, Image Sequence, and Image). The terms used in the standard are: (1) A *Project* is a scientific study that has a certain objective, defined methods, and a defined boundary in space and time. (2) A camera *Deployment* is a unique placement of a camera trap in space and time. (3) An image *Sequence* is a group of images that are all captured by a single detection event, defined as all pictures taken within 60 seconds of the previous picture or another time period defined by the Project. A sequence can either be a burst of photographs or a video clip. (4) A camera-trap *Image* is an individual image captured by a camera trap, which may be part of a multi-image sequence.

The data standard describes data relating to camera trap projects with 35 different fields across the four levels (Suppl. material 1). The *Project* section contains information about the project name, design, and objectives. Projects can either be of limited duration or be long-term monitoring. Data contributors can clearly explain limitations to data use and attribution requirements using the projectDataUseAndConstraints field. Information about the organization and people working on the project are captured in the *Project People* and the *Organization* sub-sections. The *Deployment* section contains all information related to specific locations where cameras are placed, including separate identifiers for a deployment of a camera and for deployment locations. This enables researchers to track multiple cameras that are deployed at a single location for long-term monitoring projects, as well as tracking gaps in data collection that are caused by camera or battery failure (e.g. a camera that had a 10 day gap in data collection due to battery failure would have two deploymentIDs and a cameraDeploymentBeginDateTime for the beginning of each period, but only one deploymentLocationID). The *Image* and *Sequence* sections contain data on the identification of images captured by the camera at both the detection event and image level. Every sequence or image may have multiple observations associated with it (e.g. multiple species). The *Sequence* section contains metadata for groups, or sequences, of images that are captured as part of a single detection event of an animal or group of animals. The *Images* section does the same for individual images. Depending on its goals, a given Project may record data for both Images and Sequences or just one of those categories. As modern camera traps are increasingly able to capture bursts of photos every time they are triggered some projects are classifying the animals within an entire sequence, treating the burst of photos as a single event. Other projects are interested in the data found in each photo (i.e. tracking individual animals) or have cameras that may not reliably capture bursts of photos. Both types of data are included in the CTMS to encompass this variety across projects.

The standard is compatible with the Federal Geographic Data Committee (FGDC), the Darwin Core (TDWG), the Ecological Metadata Language (EML), and the Audubon Core metadata standards to allow data to be easily cross referenced with existing data repositories, such as DataONE (Table 1).

## Discussion

**Figure 1.**
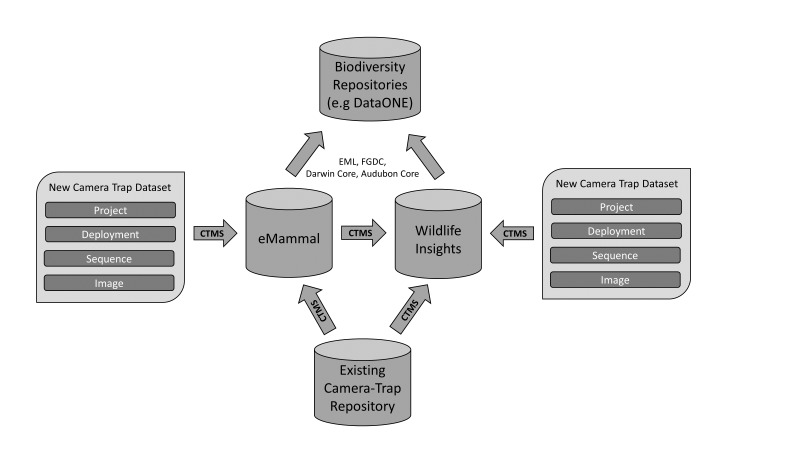
Data Sharing Using Camera Trap Metadata Standard (CTMS). A schema for data flow for researchers importing data into repositories and for data moving between repositories.

The data standard has been used to import and store data from multiple Smithsonian projects directed by different researchers, combine data from several large scale citizen-science projects (McShea et al. 2015), and to import data from multiple projects from the Wildlife Conservation Society that span several countries (www.emammal.org). The standard has also been the foundation of a successful effort to federate data from eMammal, the TEAM Network, the Wildlife Conservation Society, and Conservation International as part of the Wildlife Insights: Camera Trap Data Network (www.wildlifeinsights.org). The data standard is the basis for data sharing between Wildlife Insights and eMammal, and will allow other camera trap repositories to share data with these repositories as well (Fig. 1, ). The data standard will allow researchers to leverage the power of camera trap sampling to collect data on the distribution and abundance of a broad range of terrestrial and semi-terrestrial birds and mammals, often beyond the goals and objectives of a single research project.

Animal ecology is rapidly becoming a data-intensive science along with other branches of ecology (Hampton et al. 2013). Big data from environmental sensors is being used by ecologists to provide insight into processes that cross ecological and political boundaries, such as climate change (Kelling et al. 2009). However, for large-scale environmental data to be useful to animal ecologists we need animal occurrence datasets of matching scale (McShea et al. 2015), and this can only be accomplished by using shared data schemas to combine multiple projects (Hey et al. 2009, Michener and Jones 2012).

The data standard described here will be updated and maintained by the Wildlife Insights: Camera Trap Data Network (WI), a collaboration between the Smithsonian, the Wildlife Conservation Society, Conservation International, and the North Carolina Museum of Natural Sciences. The CTMS and associated templates (e.g., in XML and JSON, see (Suppl. material 2, Suppl. material 3) will be available at eMammal (www.emammal.org) and Wildlife Insights (www.wildlifeinsights.org) websites. The CTMS is a living document, and will maintained and improved through The Wildlife Insights: The Camera Trap Data Network, and feedback from all researchers and members of the camera trapping community is welcomed. Contact information and other information regarding Wildlife Insights may be found on the website (www.wildlifeinsights.org). The Wildlife Insights: The Camera Trap Data Network will soon provide a standard for sharing and accessing data through Application Programming Interfaces (APIs). The use of APIs will allow researchers to automatically link data between repositories of their choice that have such capabilities and allow data sharing without relying on a single camera trap data repository.

The use of standard data schemas will also allow camera trap data to be stored and archived in open data repositories, an increasingly important resource in modern ecological science (Hampton et al. 2012, Reichman et al. 2011, Michener and Jones 2012). Repositories with online access can also facilitate the discovery and use of camera trap data from around the world to advance conservation (e.g. Ahumada et al. 2013, McShea et al. 2015). This data standard is compatible with repositories for camera trap data that have recently become available to researchers and the public (www.emammal.org and www.wildlifeinsights.org). We recommend that data be stored in an online repository whenever possible to facilitate data sharing and easy access to data for both research and conservation. Interested researchers may use the eMammal or Wildlife Insights data repositories, an existing general data repository (e.g. DataONE), or create their own repository. Policies for sharing and using data from The Wildlife Insights: The Camera Trap Data Network repository website includes policies for both storing data in the repository and using publically available data from the repository. We recommend that any new repositories crosswalk their metadata structure with the CTMS to enable data sharing in the future.

The world is rapidly changing, and the pace of ecological change has outstripped the typical pace of scientific inquiry. The technologies of camera trapping and online data repositories offer a powerful tool so that scientists may provide rapid analysis and governments and conservation organizations may use this data to quickly respond to developement and change.

## Supplementary Material

Supplementary material 1 Camera Trap Metadata Standard Data type: Data Standard
Brief description: Camera Trap Metadata Standard. Standards for camera-trap data captured in 35 fields at four hierarchical levels. All data fields are cross-referenced to common ecological metadata standards where possible. Projects may use either sections 3 (image sequence data) or 4 (image data) or both, depending how data is collected.
File: oo_113263.docx Tavis D. Forrester, Tim O’Brien, Eric Fegraus, Patrick A. Jansen, Jonathan Palmer, Beth Stern, Roland Kays, Jorge Ahumada, William McShea

Supplementary material 2 XML template for camera trap metadata standard Data type: XML template
Brief description: A sample template of the camera trap metadata standard in XML.
File: oo_99335.xml Tavis D. Forrester, Tim O’Brien, Eric Fegraus, Patrick A. Jansen, Jonathan Palmer, Beth Stern, Roland Kays, Jorge Ahumada, William McShea

Supplementary material 3 JSON template for camera trap metadata standard Data type: JSON template
Brief description: A sample template of the camera trap metadata standard in JSON
File: oo_99336.json Tavis D. Forrester, Tim O’Brien, Eric Fegraus, Patrick A. Jansen, Jonathan Palmer, Beth Stern, Roland Kays, Jorge Ahumada, William McShea

## Figures and Tables

**Table 1.** Metadata Authorities and Standards

**Authority Name**	**Description**	**Link to Resource**
EAC-CPF	Encoded Archival Context for Corporations, Persons and Families	http://www2.archivists.org/groups/technical-subcommittee-on-eac-cpf/encoded-archival-context-corporate-bodies-persons-and-families-eac-cpf
Darwin Core	Data standard for describing and sharing biodiversity information.	http://rs.tdwg.org/dwc/
FGDC-Biological Profile	Describes Federal Geospatial datasets.	http://www.fgdc.gov/metadata/geospatial-metadata-standards
Ecological Metadata Language (EML)	The Ecological Metadata Language (EML) is a metadata standard developed for the ecology discipline.	http://knb.ecoinformatics.org/#external/emlparser/docs/eml-2.1.1/index.html
Audubon Core	The Audubon Core is a set of vocabularies designed to represent metadata for biodiversity multimedia resources and collections.	http://terms.tdwg.org/wiki/Audubon_Core_Structure
