# Supplementary material for: An Open Standard for Camera Trap Data
Source: Biodivers Data J. 2016 Dec 6;(4):e10197. doi: 10.3897/BDJ.4.e10197 (PMC5267527; doi:10.3897/BDJ.4.e10197)
Supplement: Supplementary material 1 — Camera Trap Metadata Standard [file biodiversity_data_journal-4-e10197-s001.docx]

**Table 1.** **Camera Trap Metadata Standard**

| **Field Name** | **Description** | | **Valid Values** | **Default Value** | **Format** | **FGDC** | **Darwin Core** | **EML Modules** | **Audubon Core** |
| --- | --- | --- | --- | --- | --- | --- | --- | --- | --- |
| **1. PROJECT** | | | | | | | | | |
| projectID | Unique identifier for the project | | Numeric or alphanumeric | NA | url or text | NA | Record-level Terms:  datasetID | EML Project | [dcterms:identifier](http://terms.tdwg.org/wiki/Audubon_Core_Term_List#dcterms:identifier) |
| projectName | Name of the project. | | NA | NA | text | Citation Information: title | Record-level Terms: collectionCode or datasetName | EML Project | [dcterms:title](http://terms.tdwg.org/wiki/Audubon_Core_Term_List#dcterms:title) |
| projectObjectives | Paragraph of text describing objectives of study. | | NA | NA | text | Identification information: abstract or purpose | Event: samplingprotocol | EML Project | [dcterms:description](http://terms.tdwg.org/wiki/Audubon_Core_Term_List#dcterms:description) |
| projectDesign | Description of study design that includes at a minimum:  - Method for selecting camera points (random, stratified, targeted, etc.)  - Paired Cameras (Y/N)  - Spacing of cameras (m)  - Any grouping or clustering of points | | NA | NA | text | Identification information: abstract or purpose | Event: samplingprotocol | EML Methods | [dcterms:description](http://terms.tdwg.org/wiki/Audubon_Core_Term_List#dcterms:description) |
| countryCode | Country the project resides in. Country code according to ISO 3166. | | ISO 3166 country code | NA | text | Identification information: placekey (place key word) | Location: country | EML Coverage | [lptc4xmpExt:CountryCode](http://terms.tdwg.org/wiki/Audubon_Core_Term_List#Iptc4xmpExt:CountryCode) |
| publishDate | Date that data is exported. | | NA | NA | date  yyyy-mm-dd | Identification Information:  pubdate | NA | EML Constraint? | NA |
| projectDataUseAndConstraints | Federation data use policy, and should also include image use constraints (including embargos). | | NA | NA | text | Identification Information: accconst  useconst | Dcterms: accessRights | EML Constraint | [xmpRights:UsageTerms](http://terms.tdwg.org/wiki/Audubon_Core_Term_List#xmpRights:UsageTerms) |
| **1.1. PROJECT PEOPLE** | | | | | | | | | |
| principalInvestigator | The first and last name of principal investigator for the project. | | NA | NA | text | Contact Information: cntpers (contact person) or origin (originator of the dataset) | Record-level terms: dcterms:rightsHolder | EML Party | [dc:creator](http://terms.tdwg.org/wiki/Audubon_Core_Term_List#dc:creator) |
| principalInvestigatorEmail | Email address for principal investigator | | Valid email | NA | text | Contact Information: cntemail (contact email) | NA | EML Party | [dc:creator](http://terms.tdwg.org/wiki/Audubon_Core_Term_List#dc:creator) |
| projectContact | The first and last name of the primary contact associated with the project. | | NA | NA | text | Contact Information: cntpos (Contact Position/title) | NA | EML Party | NA |
| projectContactEmail | Email address for project contact | | Valid email | NA | text | Contact Information: cntemail (contact email) | NA | EML Party | NA |
| **1.2. ORGANIZATION** | | | | | | | | | |
| organizationName | The name of the organization associated with a project. | |  | NA | text | *Contact Information: cnt org (contact organization) or origin (originator of the dataset) | Record-level terms: InstitutionCode (institution having custody) or OwnerInstitutionCode (owning institution) | EML Party | [dc:creator](http://terms.tdwg.org/wiki/Audubon_Core_Term_List#dc:creator) |
| **2. DEPLOYMENT** | | | | | | | | | |
| deploymentID | Unique identifier for the camera deployment (within the project) | | Numeric or alphanumeric | NA | url or text | NA | Event: eventID | EML Coverage | [ac:providerManagedID](http://terms.tdwg.org/wiki/Audubon_Core_Term_List#ac:providerManagedID) |
| cameraDeploymentBeginDateTime | Date and time that a camera was set at a location | | dateTime only | NA | dateTime  yyyy-mm-dd hh:mm:ss | Time Period Information: sngdate = caldate + time | Event: eventdate | EML Coverage | [dcterms:temporal](http://terms.tdwg.org/wiki/Audubon_Core_Term_List#dcterms:temporal) |
| cameraDeploymentEndDateTime | Date and time that a camera was removed from a location or estimated time of failure | | dateTime only | NA | dateTime  yyyy-mm-dd hh:mm:ss | Time Period Information: sngdate = caldate + time | Event: eventdate | EML Coverage | [dcterms:temporal](http://terms.tdwg.org/wiki/Audubon_Core_Term_List#dcterms:temporal) |
| deploymentLocationID | A unique name for the location of the deploymentID. This name should be consistent across projects and time periods. This is especially useful when: 1) repeat sampling occurs in the same location over different time periods and 2) camera trap projects use a multi-camera sampling scheme and 2 or more cameras may be associated with one location. | | Numeric or alphanumeric | NA | NA | Entity and Attribute Information: attr (Attribute) | NA | EML Coverage | NA |
| quietPeriod Setting | Time specified between shutter triggers when activity in the sensor will not trigger the shutter. Specified in minutes and fraction of minutes | | 0 to some maximum number | 0 | double/decimal | Methodology: methdesc (method description) | Event: samplingprotocol | EML Entity | [ac:resourceCreationTechnique](http://terms.tdwg.org/wiki/Audubon_Core_Term_List#ac:resourceCreationTechnique) |
| Latitude | Latitude of deploymentLocationID in decimal degrees using datum WGS84 | | Latitude to at least 5 decimal places | NA | double/decimal | West_Bounding_Coordinate  East_Bounding_Coordinate North_Bounding_Coordinate South_Bounding_Coordinate | Location: decimalLatitude | EML Coverage | [Iptc4xmpExt:Sublocation](http://terms.tdwg.org/wiki/Audubon_Core_Term_List#Iptc4xmpExt:Sublocation) |
| Longitude | Longitude deploymentLocationID in decimal degrees using datum WGS84 | | Longitude to at least 5 decimal places | NA | double/decimal | West_Bounding_Coordinate  East_Bounding_Coordinate North_Bounding_Coordinate South_Bounding_Coordinate | Location: decimalLongitude | EML Coverage | [Iptc4xmpExt:Sublocation](http://terms.tdwg.org/wiki/Audubon_Core_Term_List#Iptc4xmpExt:Sublocation) |
| **2.1. CAMERA PROPERTIES** | | | | | | | | | |
| cameraMake | Manufacturer and specific model of the camera | | NA | NA | text | Methodology: methdesc (method description) | Event: samplingprotocol | EML Entity | [ac:captureDevice](http://terms.tdwg.org/wiki/Audubon_Core_Term_List#ac:captureDevice) |
| **2.2. CAMERA DEPLOYMENT PROPERTIES** | | | | | | | | | |
| Bait | Type of bait used (e.g. "chicken meat") | | None  Scent  Meat  Visual  Acoustic  Other | None | text | Methodology: methdesc (method description) | Event: samplingProtocol | EML Entity | [Iptc4xmpExt:CVterm](http://terms.tdwg.org/wiki/Audubon_Core_Term_List#Iptc4xmpExt:CVterm) |
| Feature | Type of feature, if any, that camera deployment is associated with | | Road, paved  Road, dirt  Trail, hiking/people  Trail, game  Road underpass/overpass/bridge  Culvert  Burrow  Nest site  Carcass  Water source/spring  Fruiting tree  Other | Other | text | NA | Event:  fieldNotes | EML Entity | [Iptc4xmpExt:CVterm](http://terms.tdwg.org/wiki/Audubon_Core_Term_List#Iptc4xmpExt:CVterm) |
| cameraStatus | Description of camera status upon completion of deployment | | Camera Functioning  Unknown Failure  Vandalism/Theft  Memory Card/Film Failure  Camera Hardware Failure  Wildlife Damage | Camera Functioning | Text | NA | NA | EML Entity | [Iptc4xmpExt:CVterm](http://terms.tdwg.org/wiki/Audubon_Core_Term_List#Iptc4xmpExt:CVterm) |
| Other | Description of other data of interest collected at the camera deployment level | | NA | Blank | Text | NA | NA | EML Entity | NA |
| **3. IMAGE SEQUENCE (most projects will use section 3 or section 4, a few may use both)** | | | | | | | | | |
| imageSequenceID | Unique identifier for the sequence (within the project) | | Numeric or alphanumeric | NA | url or text | NA | NA | EML Entity | [ac:providerManagedID](http://terms.tdwg.org/wiki/Audubon_Core_Term_List#ac:providerManagedID) |
| ImageSequenceBeginTime | Date and time of the first picture in the sequence | | dateTime only | NA | dateTime yyyy-mm-dd hh:mm:ss | Time Period Information: sngdate = caldate + time | Event:  eventDate  eventTime | EML Entity | [xmp:CreateDate](http://terms.tdwg.org/wiki/Audubon_Core_Term_List#xmp:CreateDate) |
| ImageSequenceEndTime | Date and time of the last picture in the sequence | | dateTime only | NA | dateTime yyyy-mm-dd hh:mm:ss | Time Period Information: sngdate = caldate + time | Event:  eventDate  eventTime | EML Entity | [xmp:CreateDate](http://terms.tdwg.org/wiki/Audubon_Core_Term_List#xmp:CreateDate) |
| sequenceIdentifiedBy | First and last name or identifier of the person who identified and counted the animals in the photo sequence | | NA | NA | text | Taxonomic System:  Identifier: Contact Person Primary | Identification: identifiedBy | EML Entity | [dwc:identifiedBy](http://terms.tdwg.org/wiki/Audubon_Core_Term_List#dwc:identifiedBy) |
| imageSequenceDefinition | The amount of time (in seconds) that defines the grouping of the sequence. All pictures that are separated by less time than this value are grouped into one sequence | | Numeric | None | non negative integer | NA | NA | EML Entity | NA |
| **3.1. IMAGE SEQUENCE ANIMAL** | | | | | | | | | |
| ImageSequenceCount | The number of individual animals of a particular species in the sequence | | numeric | NA | non negative integer | NA | Occurrence:  organismQuantity  organismQuantityType | EML Entity | NA |
| speciesScientificName | Latin name for a species (Genus and species) in the sequence | | IUCN Red List for mammals and Birdlife International for birds | NA | text | Taxonomy: taxonrn and taxonrv (taxon rank name and value) | Taxon: Scientificname | EML Entity | [dwc:scientificName](http://terms.tdwg.org/wiki/Audubon_Core_Term_List#dwc:scientificName) |
| **4. IMAGES (most projects will use section 3 or section 4, a few may use both)** | | | | | | | | | |
| imageID | Unique identifier for the image (within the project) | | Numeric or alphanumeric | NA | url or text | NA | NA | EML Entity | [ac:providerManagedID](http://terms.tdwg.org/wiki/Audubon_Core_Term_List#ac:providerManagedID) |
| dateTimeCaptured | Date and time an image was taken | | dateTime only | NA | dateTime yyyy-mm-dd hh:mm:ss | Time Period Information: sngdate = caldate + time | Event: eventdate | EML Entity | [xmp:CreateDate](http://terms.tdwg.org/wiki/Audubon_Core_Term_List#xmp:CreateDate) |
| photoType | Type of photo that was taken (e.g., Animal, Set-up) | | Start  End  Set Up  Blank  Animal  Staff  Unknown  Unidentifiable  Timelapse | NA | text | Identification Informaton: themekey (theme keywords) | Occurrence: occurrenceRemarks | EML Entity | [Iptc4xmpExt:CVterm](http://terms.tdwg.org/wiki/Audubon_Core_Term_List#Iptc4xmpExt:CVterm) |
| photoTypeIdentifiedBy | First and last name of the person who identified the photo type. | | NA | NA | text | Taxonomic System:  Identifier: Contact Person Primary | Identification: identifiedBy | EML Entity | [dwc:identifiedBy](http://terms.tdwg.org/wiki/Audubon_Core_Term_List#dwc:identifiedBy) |
| **4.1. IMAGE ANIMAL** | | | | | | | | | |
| imageCount | The number of individual animals of a given species in the image | numeric | | NA | non negative integer | NA | Occurrence: individualCount | EML Entity | NA |
| speciesScientificName | Latin name for a species (Genus and species) in the image | IUCN Red List for mammals and Birdlife International for birds | | NA | text | Taxonomy: taxonrn and taxonrv (taxon rank name and value) | Taxon: Scientificname | EML Entity | [dwc:scientificName](http://terms.tdwg.org/wiki/Audubon_Core_Term_List#dwc:scientificName) |

Standards for camera-trap data captured in 35 fields at four hierarchical levels. All data fields are cross-referenced to common ecological metadata standards where possible.
